# Comparison of the hemodynamic changes between preeclamptic and normotensive parturients who underwent cesarean section under spinal anesthesia at North Showa zone public hospitals, Oromia region, Ethiopia, 2022: a prospective cohort study

**DOI:** 10.1186/s12871-023-02314-7

**Published:** 2023-11-25

**Authors:** Bizuwork Girma Belachew, Blen Kassahun Dessu, Birhanu Wondimeneh Demissie, Ashagrie Sintayhu, Getahun Dendir, Abas Ali, Redi Awol, Dugo Angasa, Asaminew Tasew, Oliyad Eshatu, Aschalew Besha Desta, Derara Girma, Getachew Debalke

**Affiliations:** 1 School of Anaesthesia, Wolaita Sodo University, Wolaita Sodo, Ethiopia; 2 School of Nursing and Midwifery, Addis Ababa University, Addis Ababa, Ethiopia; 3 Faculty of Health, School of Nursing & Midwifery, University of Technology Sydney, Sydney, Australia; 4 Department of Anaesthesia, Worabe University, Worabe, Ethiopia; 5 Department of Anaesthesia, Hawasa University, Hawasa, Ethiopia; 6 Department of Anaesthesia, Ambo University, Ambo, Ethiopia; 7 Department of Anaesthesia, Hawasa University, Hawasa, Ethiopia; 8 Public Health Department, College of Health Sciences, Salale University, Fiche, Ethiopia; 9 Department of Anaesthesia, College of Health Sciences, Arsi University, Asella, Ethiopia

**Keywords:** Hemodynamic, Spinal anaesthesia, Preeclampsia, Caesarean section

## Abstract

**Background:**

Spinal anaesthesia complicates maternal hemodynamic and may expose the parturient to dangerous cardiovascular problems. Up to 7% to 89.2% of pregnant women can suffer from spinal anaesthesia-related hypotension. The aim of this study to compare the hemodynamic changes between preeclamptic and normotensive parturients who underwent caesarean section under spinal anaesthesia at North Showa Zone Public Hospitals, Oromia Region, from February 15 to May 15, 2022.

**Methods:**

A prospective cohort study was conducted on a total of 140 parturients (70 in each group) who underwent cesarean delivery under spinal anesthesia. The study participants were chosen using a consecutive sampling technique**.** Data were collected from patient charts and intraoperative observations and entered into the Epi Data software version 4.6 and exported to the Statistical Package for the Social Sciences version 25 software. Hemodynamic change = (baseline value-current value/baseline value) * 100. The independent t-test, Mann–Whitney U test, two ways mixed ANOVA, chi-square, and Fisher's exact test was used to analyze the data as appropriate. A *P* < 0.05 was statistically significant.

**Results:**

The mean percentage change in SBP, DBP, and MAP after spinal anaesthesia was a statistically significant difference between the normotensive and preeclamptic groups, except MAP at 15 min was comparable between the two groups with *p* = 0.638. The proportion of preeclamptic parturients who develop hypotension was 47%, compared to 74% of normotensive parturients, and the RR of developing hypotension, if participants were preeclamptic, was 0.63, with a 95% confidence interval of 0.412 to 0.978 and a *p* = 0.039. The mean change in heart rate during the first 15 min was comparable between the groups.

**Conclusion:**

In contrast to normotensive parturients undergoing caesarean section under spinal anaesthesia, our study found that the hemodynamic change was lower in preeclamptic parturients. The proportion of preeclamptic women who develop hypotension was 47%, compared to 74% of normotensive parturients.

## Background

Spinal anesthesia is a popular anesthesia technique for cesarean sections because it avoids the risks associated with general anesthesia, such as difficult intubation and aspiration of gastric contents [1, 2]. The benefits of spinal anesthesia include its simplicity, rapid onset, low failure rate, low drug dose, and profound or dense sensory and motor block [3].

A drop in mean arterial pressure (MAP) of more than 20% from baseline is considered hypotension following spinal anesthesia [4]. Despite several attempts to reduce both the incidence and severity of post-spinal hypotension, it remains common. The primary cause of hypotension after spinal anesthesia is a decrease in systemic vascular resistance due to vasodilation caused by the blockade of preganglionic sympathetic fibers [2, 5, 6].

Cesarean delivery complicates maternal hemodynamics and may expose the parturient to dangerous cardiovascular problems. Spinal anesthesia-related hypotension affects up to 7% to 89.2% of pregnant women after cesarean delivery [3, 7]. Nausea, vomiting, and light-headedness are common symptoms of spinal-induced maternal hypotension. It may also reduce utero placental blood flow, leading to fetal acidosis [8].

Despite the fact that spinal anesthesia is frequently the favored approach for caesarean birth [1, 9], due to considerable intravascular volume deficits caused by broad arteriolar vasoconstriction, it was previously not chosen for severely preeclampsia women. During obstetric anesthesia, maintaining hemodynamic stability is great importance to anesthesiologists, especially women with preeclampsia childbirth planning to have a caesarean section(CS) [7, 10]. In the United States the case fatality rate from regional anesthesia for caesarean section was 38 per million anesthetics [11]. Whereas the obstetric mortality due to spinal anesthesia hypotension, bradycardia, nausea and vomiting, high spinal anesthesia, and cardiac arrest can be avoided with proper training and resources [12–14].

Furthermore, the incidence of spinal anesthesia-induced maternal hypotension varied among research, making it impossible to establish standard guidelines and create a local therapeutic plan [15–17]. Other investigations found no differences between severely preeclampsia and healthy parturient in the incidence of hypotension, degree of hemodynamic change, or neonatal prognosis [18]. Previous studies have shown inconsistent findings on the incidence of hypotension between preeclampsia and normotensive patients [19, 20].

In 2015, Sub-Saharan Africa accounted for two-thirds, of all maternal deaths worldwide. From the two thirds of deaths, the magnitude of spinal anesthesia related deaths is around 6% [21]. Despite a considerable shift in practice from general to spinal anesthesia for caesarean sections in South Africa, preventable anesthesia related deaths still occur. During the three-year period 2011–2013, spinal anesthesia was involved in 71% of the 105 anesthesia-related deaths, the majority of which were considered avoidable [22]. However, maternal hypotension caused by spinal anesthesia remains the most common problem [23, 24]. The most common complication related to maternal morbidity and mortality during cesarean section was hypotension following spinal anesthesia [25]. When preeclampsia patients have Cesarean section under spinal anesthesia, they are thought to be at a higher risk of significant hypotension [26].

## Methods

### Study design and areas

An institutional-based prospective cohort study was carried out at public hospitals in North Showa Zone, Oromia region, from the seven public hospitals in the North Showa zone, those three hospitals, Salale University Comprehensive Specialized Hospital (SUCSH), Kuyu General Hospital (KGH), and Chancho Hospital, were selected by simple random sampling. SUCSH, which is located 112 km from Addis Ababa, KGH, which is located 43 km from SUCSH and 165 km from Addis Ababa, and Chancho Hospital, which is located 40 km from the capital city of Ethiopia.

Ethical approval for this study was obtained from the ethical review board of Wolaita Sodo University, College of Health Sciences and Medicine, with protocol unique No. CHSM/ERC/03/14. Then, a support letter was obtained from the Medical Director of the hospital to conduct the research, and written informed consent was obtained from study participants. Privacy and confidentiality of the interviews and information gathered was assured at all level of study.

All Parturients who underwent caesarean section delivery under spinal anesthesia at public hospitals in the Oromia Region, North Showa zone served as population’s source. ASA physical status II and III parturients, women over 18 years old, and women carrying a singleton pregnancy were included in the study from February 15 to May 15, 2022. Those who are in active labor, Patients with chronic hypertension, renal, or cardiac disease, diabetes, hypothyroidism, or hyperthyroidism in a parturient, Parturients with placenta abruption, placenta previa, BMI > 35 kg/m2, and adjuvants added to local anesthesia were excluded from the study.

### Sample size and sampling technique

The sample size was calculated using G*power version 3.1.9.7 software and the mean with standard deviation between the two groups were taken from a previous study done in Gondar on the topic of hemodynamic changes after spinal anesthesia in preeclamptic patients undergoing cesarean section [27, 28]. Based on this study, the lowest decrease of DBP in healthy groups is 26.18% ± 4.07 and the lowest decrease of DBP in preeclamptic groups is 23.93% ± 4.79. Using power of 80%, alpha 0.05, and effect size 0.5062319 the sample size was calculated using a prior power analysis with G Power 3.1.9.7 software to be 126. An additional 10% was added to the enrollment to offset potential dropouts, assuming the balanced design. The total sample size increases to 140 participants, with 70 in each group. A consecutive sampling technique was used to select study participants based on our inclusion criteria. The sample size was allocated to the hospitals by proportional to size using situational analysis from last year's performance report from selected hospitals.

### Data collection procedure

Three trained BSc anesthetists used structured check lists and questionnaires developed by reviewing various literature to collect data from patient charts and intraoperative observations. Before the actual data collection, the adapted format was evaluated by an expert researcher. Patients were monitored with non-invasive automated blood pressure cuffs, an ECG, and pulse oximetry. Before any invasive procedure or spinal anesthesia, baseline blood pressure and heart rate were measured. All patients were preloaded with crystalloids (500–1000 ml) 10–15 min prior to SA being given, with oxytocin 10–20 IU, and then 10–12.5 mg of 0.5% isobaric bupivacaine was given between lumbar three and four and lumbar four and five levels. Immediately after spinal anesthesia administration, the parturients were put in a supine position with a pillow inserted under their shoulder, and the maternal hemodynamics parameters (SBP, DBP, MAP, and HR) were recorded. The exposed group was those with a systolic blood pressure (SBP) of greater than 140 mm Hg and a diastolic blood pressure (DBP) of greater than 90 mm Hg using noninvasive automated blood pressure (NIBP) measurement and urine protein dipstick plus one or two, and the controlled group was those with normal blood pressure and no protein urea (trace or negative protein dipstick). All patients selected for the study were asked for their consent, and after a complete sensory (> T10) block, a skin incision was started. Assessments of hemodynamic parameters were made at three-minute intervals, initially for the first twenty minutes, then at five-minute intervals until the end of the procedure. Supervisors checked each questionnaire daily, with a further cross-check by the principal investigator for completeness and consistency of data.

#### Data processing and analysis

The data was checked for completeness, coded, and entered into Epi Data software version 4.6 before being exported into Statistical Package for the Social Sciences (SPSS) version 25 software, where missing values were checked before analysis.

A mean with a standard deviation was used to summarize data, tables, and figures to display results. The data were tested for normality using the Shapiro–Wilk normality test and histogram inspection, and homogeneity of variance was assessed using Levene’s test for equality of variances of two samples in SPSS version 25 software. Outliers were assessed by box plots. Normal distributed continuous variable was compared by using the independent t test and expressed by mean ± SD. Non normal distributed continuous data was compared using the Mann–Whitney U-test and expressed as the median or interquartile range (IQR).

The Chi-square test, or Fisher exact test, was used to calculate the categorical data between two groups as appropriate. The relative risk (RR) with a 95% confidence interval (CI) was used to determine the incidence of hypotension. A P value of less than 0.05 is considered significant. For repeated measures, homogeneity of covariance was assessed by Box’s M test. Mauchly’s test was used to check for sphericity, and the Greenhouse–Geisser correction was used when the assumption of sphericity was violated for the two-way interaction.

#### Operational definitions

*Preeclampsia* is defined as a systolic blood pressure (SBP) of greater than 140 mm Hg, a diastolic blood pressure (DBP) of greater than 90 mm Hg using noninvasive automated blood pressure (NIBP) measurement and urine protein dipstick plus one or two, which is recorded from the patient's chart [29].

*Severe preeclampsia* is defined as systolic blood pressure (SBP) being greater than 160 mm Hg, diastolic blood pressure (DBP) being greater than 110 mm Hg using noninvasive automated blood pressure measurement (NIBP) and urine protein dipstick plus two or more, which is recorded in the patient's chart [29].

*Hemodynamic change* is defined as blood pressure and heart rate change from baseline by 20%, either an increase or decrease [7, 30, 31].

*Mild hypotension*: less than 20% decline in mean arterial blood pressure (MAP) below the base line in both groups [32].

*Moderate hypotension*: Blood pressure reduction of 20% to 30% from the mean arterial blood pressure baseline [32–34]

*Severe hypotension*: more than 30% decline in mean arterial blood pressure below the base line in both groups [32, 33].

The percentage fall in blood pressure and heart rate from base line (SBP, DBP, and MAP and HR) between two measurements is calculated as:

Percentage of hemodynamic change = (baseline value-current value/baseline value) * 100.

*Negative (-) values* indicate the percent fall of a parameter (SBP, DBP, MAP) from its corresponding baseline value and are higher than baseline values at some intervals after spinal anesthesia.

*Normotensive*: Having normal blood pressure and have no protein urea (trace or negative protein dipstick) [35].

## Results

### Demographic and maternal related data between the two groups

One hundred forty parturients who underwent cesarean sections and fulfilled inclusion criteria were enrolled into two groups (70 in each group). In terms of age, weight, height, BMI, new-born weight, and parity, there was no significant difference between the two groups (Table 1).

**Table 1:** Demographic and maternal related data between preeclamptic and normotensive groups at North Showa Zone Public Hospitals, Oromia Region; 2022

Variables	Preeclamptic group(*n* = 70)	Normotensive Group(*n* = 70)	Comparison		
	Mean ± SD	Mean ± SD	t value	95% CI	*P* value
Age (yr.)	27.46 ± 5.62	26.53 ± 4.62	1.06	- 0.79 to 2.65	0.288
Weight(kg)	64.41 ± 6.02	64.67 ± 5.36	- 0.28	- 2.17 to 1.63	0.776
Height(m)	1.59 ± 0.06	1.60 ± 0.07	- 0.92	- 0.03 to 0.01	0.358
Body Mass Index(kg/m2)	25.66 ± 3.18	25.17 ± 2.52	1.01	- 0.46 to 1.45	0.310
Gestational age (week)	36.56 ± 0.879	37.89 ± 0.776	–6.73	-1.22 to -0.66	0.001^*^
Hemoglobin (g/dl)	12.35 ± 0.92	12.35 ± 0.84	- 0.01	- 0.29 to 0.29	0.985
Baby weight(kg)	3.31 ± 0.21	3.39 ± 0.25	- 1.91	- 0.15 to 0.02	0.058
Parity	0.87 ± 1.05	1.2 ± 1.06	- 1.86	- 0.68 to 0.02	0.067

^*^Independent t-test statistically significant (Mean ± SD)

The majority of preeclamptic parturients were ASA III and the remaining were ASA II, while, all parturients in the normotensive group were ASA II, and this difference was statistically significant between groups; *p* = 0.001 (Table 3). The preeclamptic group had a significantly lower mean gestational age at the time of cesarean section: 37.89 0.776 weeks in the normotensive group versus 36.56 0.879 weeks in the preeclamptic group, with a 95% CI of -1.22 to -0.66 and a p value of 0.001 and a t value of (138) = -6.73.

The effect of ASA status and gestational age on the hemodynamic change for the preeclamptic group was adjusted to an odd ratio of 0.660 with a 95% CI of 0.248 to 1.753 and a *p* value of 0.404, which was non-significant. The adjusted odd ratio for gestational age was 0.905 with a 95% CI of 0.519 to 1.578 and a p value of 0.726, which was statistically non-significant. The adjusted odd ratio for the normotensive group of gestational age was 1.494 with a 95% CI of 0.709 to 3.151 and a *p* = 0.291, which was non-significant (Table 1).

### Mean baseline hemodynamic, anesthetic and intraoperative data between the two groups

In terms of the mean preload fluid, baseline HR, dose of bupivacaine, dose of oxytocin, speed of bupivacaine administration, level of sensory block, surgeon status, and experience of anesthetist, these were comparable in both groups (Tables 2 and 3). The mean baseline MAP in the preeclamptic group was 108.64 ± 7.20, while in the normotensive group it was 100.24 ± 7.08 with a 95% CI of 5.01 to 9.78 and a *P* = 0.001 and t (138) = 6.12.

**Table 2:** Mean baseline hemodynamic and intraoperative data of parturients between preeclamptic and normotensive groups at North Showa Zone Public Hospitals, Oromia Region, Ethiopia, 2022

Variables	Preeclamptic group(*n* = 70)	Normotensive group(*n* = 70)	Comparison		
	Mean ± SD	Mean ± SD	t value	95% CI	*P* value
Crystalloid fluid preloading ( ml)	625 ± 130.16	641 ± 109.07	-0.80	- 56.70 to 23.84	0.421
Baseline MAP (mmHg)	108.64 ± 7.20	100.24 ± 7.08	6.12	5.01 to 9.78	0.001*
Baseline HR (beat/minute)	96.87 ± 12.56	96.36 ± 4.95	0.62	- 1.12 to 2.14	0.535
Estimated blood loss (ml)	580.94 ± 51.2	611.46 ± 50.38	- 3.56	-47.46 to–13.56	0.001*
Dose of bupivacaine (mg)	12.22 ± 0.75	12.02 ± 0.97	1.35	- 0.09 to 0.49	0.177
Speed of bupivacaine given (ml/sec)	10.11 ± 3.26	10.51 ± 3.84	- 0.66	- 1.59 to 0.79	0.508
Dose of oxytocin ( IU)	12.71 ± 4.79	12.29 ± 4.23	- 0.56	- 1.08 to 1.93	0.576
Experience of anesthetist (year)	2.91 ± 0.28	2.84 ± 0.36	1.29	- 0.03 to 0.18	0.199
Duration of surgery ( minute)	26.64 ± 2.51	30.00 ± 3.18	- 6.98	- 4.31 to -2.39	0.001*

^*^Independent t-test statistically significant (Mean ± SD)

**Table 3:** Non parametric data between preeclamptic and normotensive groups at North Showa Zone Public Hospitals, Oromia Region; 2022

Variables		Preeclamptic group(*n* = 70)	Normotensive group(*n* = 70)	*P* value
ASA status n (%)				
ASAII		31(44.29%)	70(100%)	0.001*
ASA III		39(55.71%)		
Intraoperative IV crystalloid fluid (ml)		1000(500)	1900(550)	0.001*
Level of sensory block		T5(T5)	T5(T7)	0.334
Surgeon status n (%)	Gynecologist	53(76%)	17(26%)	1.000
	IESO	17(24%)	53(74%)	

*Median (IQR)* Mann Whitney U test, *Frequency (%)* Fisher exact test & chi-square test

^*^Significant

In terms of intraoperative fluid consumption, there was a statistically significant difference between the groups. The median intra-operative crystalloid requirement in the preeclamptic group was 1000(500) ml, while in the normotensive group it was 1900(550) ml (*P* = 0.001) (Table 3). The estimated blood loss in the preeclamptic group was significantly lower, with a mean 580.94 ± 51.2, while in the normotensive group it was 611.46 ± 50.38 and a 95% CI of -47.46 to -13.56 with a *p* = 0.001 (Table 2).

### Patterns of change in hemodynamic parameters between the two groups

#### Mean changes in heart rate between the groups

The mean change in heart rate between the two groups was no different from the start of SA to first15 minutes. The mean change in heart rate from 18 to 31 min was a statistically significant difference between the groups (Table 4).

**Table 4:** Mean changes in HR data between the two groups at North Showa Zone Public Hospitals, Oromia Region; 2022

Variable	Normotensive Group	Preeclamptic group	Comparison		
	Mean ± SD	Mean ± SD	t value	95% CI	*P* value
Base line HR ( beat/minute)	96.36 ± 4.95	96.87 ± 12.56	0.62	- 1.12 to 2.14	0.535
Immediate after SA HR	94.10 ± 9.59	91.04 ± 11.00	- 1.75	- 6.50 to 0.39	0.082
At 3 HR	87.94 ± 20.2	87.8 ± 16.00	0.04	- 5.94 to 6.23	0.615
At 6 HR	88.63 ± 16.00	84.37 ± 14.99	1.62	- 0.93 to 9.44	0.107
At 9 HR	86.24 ± 11.67	84.3 ± 9.43	1.08	- 1.60 to 5.49	0.281
At 12 HR	85.13 ± 15.13	80.67 ± 13.34	1.84	- 0.31 to 9.22	0.067
At 15 HR	83.63 ± 10.07	81.93 ± 8.36	1.08	- 1.39 to 4.79	0.297
At 18 HR	86.09 ± 6.97	79.60 ± 6.31	- 5.76	- 8.71 to – 4.26	0.001*
At 21 HR	83.61 ± 5.89	77.13 ± 5.76	- 6.57	- 8.43 to – 4.53	0.001*
At 26 HR	84.21 ± 5.55	79.09 ± 5.72	- 5.38	- 7.01 to – 3.24	0.001*
At 31 HR	76.48 ± 9.01	86.11 ± 7.65	5.71	6.29 to 12.98	0.001*

*Mean* ± *SD* Independent t test, *HR* heart rate, *SA* spinal anesthesia

^*^Significant

#### Mean changes in MAP between the two groups

The mean change in MAP after spinal anesthesia between the groups was statistically significantly different between normotensive and preeclamptic groups with a *p* value less than 0.05, except at 15 min. At 15 min, the mean change in MAP was non-significant, with a 95% CI of -4.00 to 2.46, a *p* value of 0.638, and t (138) = -0.47 (Table 5).

**Table 5:** Mean changes in MAP data between the two groups at North Showa Zone Public Hospitals, Oromia Region; 2022

Variable at different time	Normotensive Group	Preeclamptic group	Comparison		
	Mean ± SD	Mean ± SD	t value	95% CI	*P* value
Base line MAP (mmHg)	100.24 ± 7.08	108.64 ± 7.20	6.12	5.01 to 9.78	0.001*
Immediate after SA MAP	83.64 ± 9.56	95 ± 8.68	7.35	8.30 to 14.40	0.001*
At 3 MAP	79.61 ± 9.19	91.97 ± 7.81	8.57	9.50 to 15.20	0.001*
At 6 MAP	77.6 ± 6.83	87.96 ± 6.86	8.94	8.06 to 12.64	0.001*
At 9 MAP	76.93 ± 8.24	87.14 ± 8.37	7.27	7.43 to 12.99	0.001*
At 12 MAP	77.94 ± 7.47	90.37 ± 8.54	9.16	9.76 to 15.11	0.001*
At 15 MAP	79.23 ± 9.20	78.46 ± 10.11	- 0.47	- 4.00 to 2.46	0.638
At 18 MAP	75.73 ± 7.12	83.51 ± 7.31	6.38	5.37 to 10.19	0.001*
At 21 MAP	92.73 ± 7.04	83.66 ± 6.85	- 7.72	-11.39 to -6.74	0.001*
At 26 MAP	75.36 ± 7.05	89.44 ± 7.48	11.46	11.65 to 16.51	0.001*
At 31 MAP	74.95 ± 5.97	89.89 ± 5.51	12.85	12.62 to 17.24	0.001*

*Mean* ± *SD* Independent t test, *MAP* mean arterial pressure, * Significant

#### Mean changes in SBP and DBP between the two groups

**Fig. 1:**
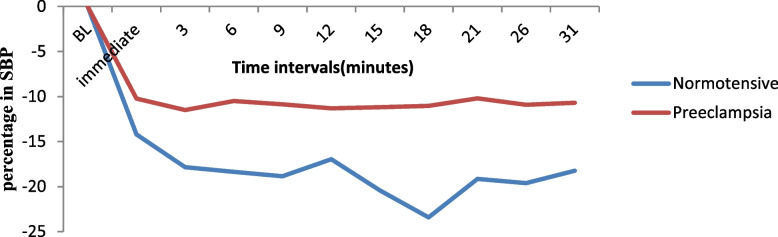
The mean percent change in SBP from baseline between the two groups at North Showa Zone Public Hospitals in Oromia Region, Ethiopia, 2022

The mean change in SBP and DBP after spinal anesthesia to the end of surgery in both groups decreased from the baseline, but there were more changes in the normotensive groups. The mean change in SBP and DBP after spinal anesthesia between the groups was statistically significantly different between normotensive and preeclamptic groups with a p value less than 0.05 at all levels of time. The lowest percent decrease in SBP from baseline SBP was 23.42% ± 7.60 for normotensive groups, and 11.50% ± 8.98 was the lowest decrement for preeclamptic groups (Fig. 1).

**Fig. 2:**
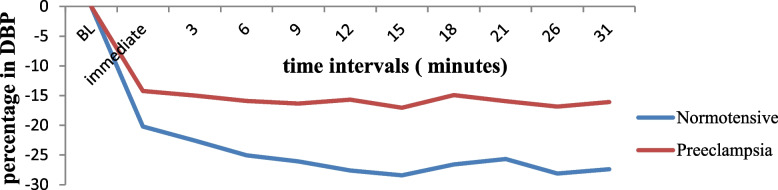
The mean percent change in DBP from baseline between the two groups at North Showa Zone Public Hospitals in Oromia Region, Ethiopia, 2022

The lowest percent decrease in DBP from baseline DBP was 28.42% ± 9.64 for the normotensive group, and 17.05% ± 10.98 was the lowest decrement for the preeclamptic group, with a 95% CI of -14.824 to -7.915 and a *p* value of 0.001 and t (138) = -6.50 (Fig. 2). There was statistically significant difference between the two groups from immediate of SA to the 31 min.

#### Comparison of repeated measures between /within groups on mean arterial blood pressure measurements

**Fig. 3:**
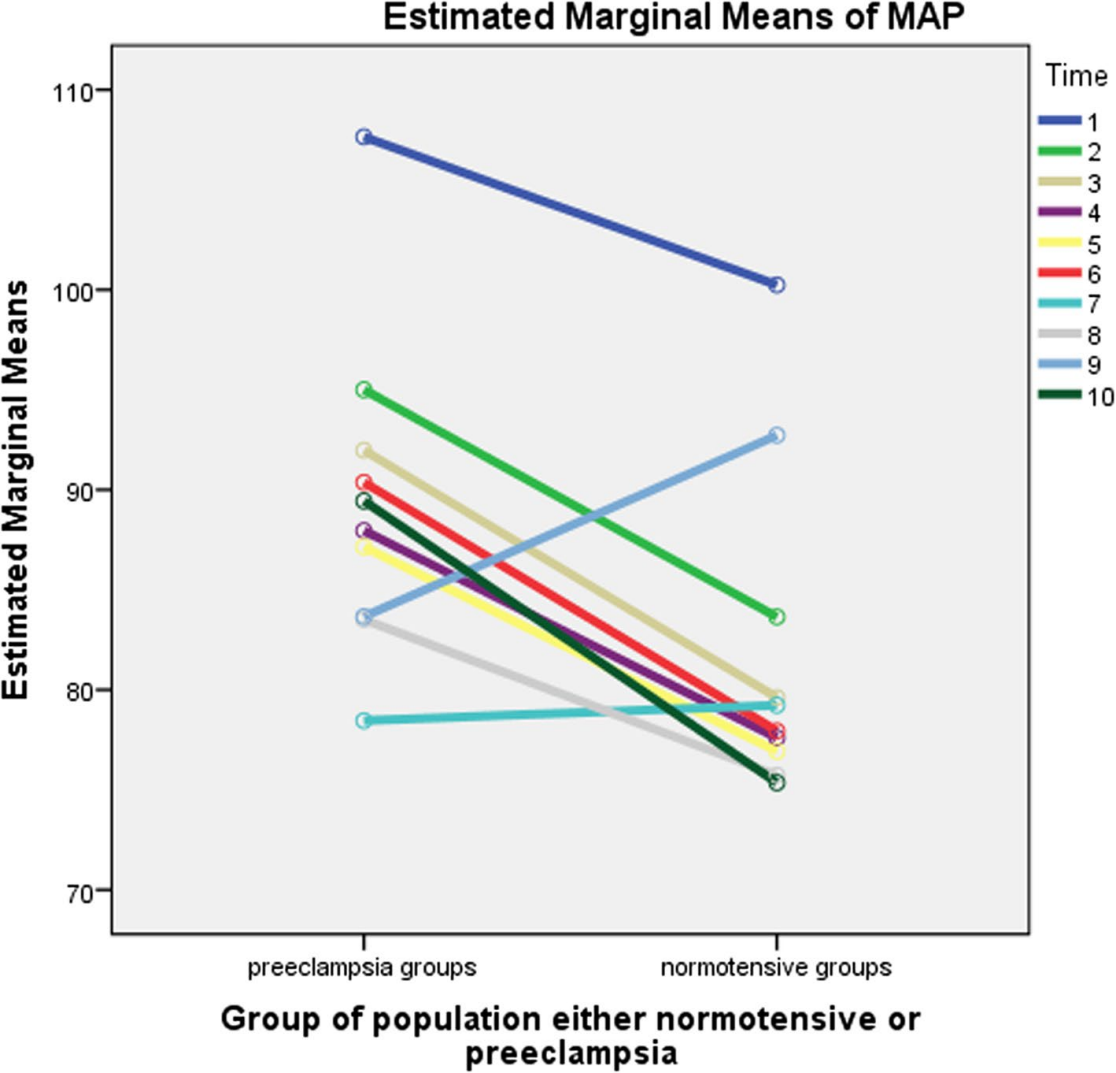
Comparison of the repeated measures between /within groups on MAP measurements at North Showa Zone Public Hospitals in Oromia Region, Ethiopia, 2022

The group and time had a statistically significant interaction on MAP F (8.007, 1104.968) = 38.833; *P* = 0.001, partial eta squared = 0.220, = 0.831. The group main effect revealed a statistically significant difference in mean MAP between exposed groups: F (1, 138) = 94.180; *P* = 0.001; and Partial eta squared = 0.406. The main effect of time is a statistically significant difference in mean MAP at the different time points: F (8.007, 1104.968) = 157.568; *P* = 0.001, and partial eta squared = 0.533; ε = 0.831. There was no statistically significant interaction between the baseline and other levels of time on MAP for the groups (*p* > 0.05). When compared to preeclamptic groups, the mean change in MAP, the more decrement, was seen in normotensive groups except at 15 and 21 min of SA (Fig. 3).

### Incidence of hypotension between the two groups

The proportion of preeclamptic women who develop hypotension was 47%, compared to 74% of normotensive women, and the RR of developing hypotension if participants were preeclamptic was 0.63, with a 95% confidence interval of 0.412 to 0.978 and a *p* = 0.039. The preeclamptic group has a 37% lower risk of developing hypotension than the normotensive parturient group.

**Fig. 4:**
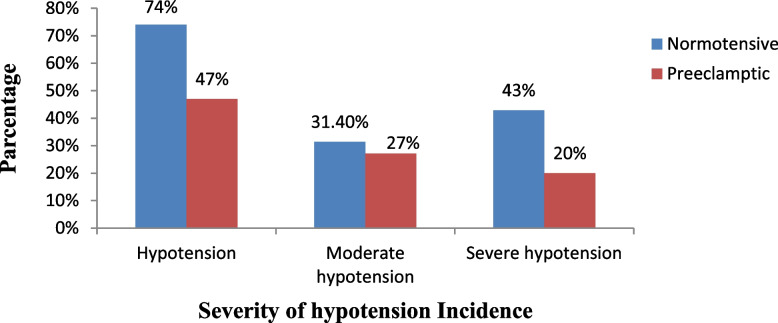
Data on the severity of hypotension incidence in the two groups at North Showa Zone Public Hospitals in Oromia Region; 2022

The proportion of preeclamptic women who develop moderate hypotension was 27%, compared to 31.4% of normotensive women, and the RR of developing moderate hypotension if participants were preeclamptic was 0.86, with a 95% confidence interval of 0.467 to 1.594 and a p = 0.6440. The proportion of preeclamptic women who develop severe hypotension was 20%, compared to 43% of normotensive women, and the RR of developing severe hypotension if participants were preeclamptic was 0.46, with a 95% confidence interval of 0.251 to 0.866 and a *p* = 0.0161 (Fig. 4).

**Fig. 5:**
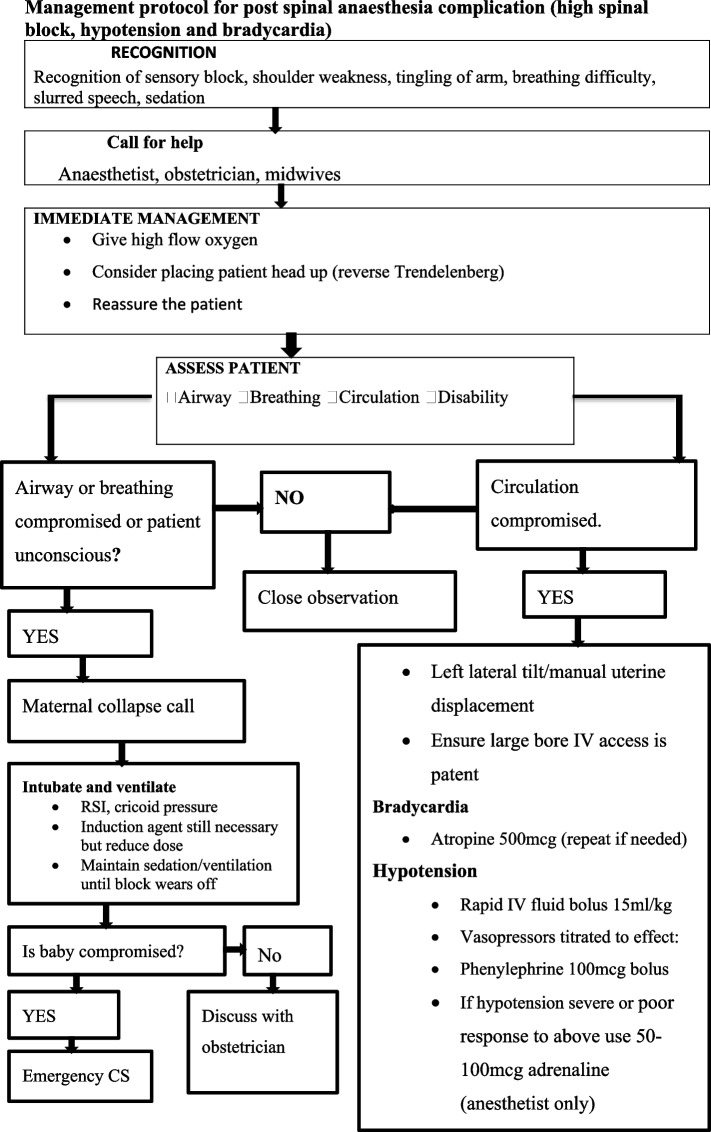
Management protocol for post spinal anaesthesia complication

Hypotension was quickly treated by increasing the fluid administration rate and administering atropine and vasopressors (Fig. 5). Around 20 patients in the normotensive group received an IV bolus of vasopressor for hypotension treatment, while 8 patients in the preeclamptic group received vasopressor. The incidence of moderate hypotension was comparable between the two groups, but there was a statistical difference in the incidence of hypotension and severe hypotension.

## Discussion

The demographic and maternal variables of parturients were comparable between both groups with *p* > 0.05. Our study found that the proportion of preeclamptic women who develop hypotension was 47%, compared to 74% of normotensive parturients. Once spinal anesthesia was induced, SBP, DBP, and MAP fell in both groups, but more so in the normotensive groups than in the preeclamptic parturients, and statistically significant difference between the two groups with *p* < 0.05 except MAP at 15 min was comparable between the two groups. The change in mean heart rate between the two groups during the first 15 min it was comparable with a *p*- value > 0.05. However, beyond those 18 min, there was a significant difference between the groups.

The normotensive group’s higher vasopressor consumption than that of preeclampsia; around 29% of normotensive parturients took vasopressor but around 11% in preeclamptic groups with *p* = 0.024. The normotensive groups had significantly higher mean duration of surgery and more blood loss than those of preeclamptic parturients with *p* < 0.05. In the normotensive group, the median intra-operative crystalloid demand was 1900(550) ml, compared to 1000(500) ml in the preeclamptic group (*P* = 0.001).

In the Republic of Macedonia, a prospective study by Sivevski et al. found that the incidence of hypotension was 25% in preeclamptics compared to 53% in healthy parturients. That blood pressure (SBP, DBP, and MAP) falls (%) from baseline was significantly greater in healthy parturients than in those with preeclampsia [36]. This research supported our findings.

Significant variations in SBP, DBP, and MAP were reported in both groups at each time point by Nikooseresht et al. [37]. This research supported our findings.

In spite of receiving fewer crystalloid fluids and a larger dose of 0.5% bupivacaine, a prospective comparative study of 60 parturients by Aya et al. revealed that the incidence of hypotension in severely preeclamptic parturients was 16.6% and 53.3% in normotensive groups. Significant hypotension was defined as a systolic blood pressure reduction of less than 100 mmHg in healthy pregnant women or a mean blood pressure decrease of 30% in both groups [23]. Following spinal anesthesia, the normotensive groups had larger percentage decreases in mean arterial and diastolic pressures (from baseline) than the preeclamptic groups. This research supported our findings. The slight differences in the incidence of hypotension may be partly due to their study’s low dose of bupivacaine (10 mg vs. the mean of 12.04 mg).

According to Chowdhury et al.’s study, Preeclamptic patients had a lower risk of hypotension than healthy parturients (*P* < 0.05). Overall, intravenous phenylephrine dosages for treating hypotension were generally less in pre-preeclampsia patients. In terms of age, weight, height, and volume of ringer lactate preloading, patients with preeclamptic and normotensive patients were equivalent [38]. This study's findings were similar to ours finding.

In India in 2016, subarachnoid block was associated with better perioperative hemodynamic stability, less hypotension, and less vasopressor consumption in preeclampsia groups [31]. In our study, preeclamptic groups had less vasopressor consumption than normotensive groups. This research supported our findings. According to Ishrat and Raja's study, preeclamptic patients had less hypotension after spinal anesthesia than healthy pregnant mothers, while healthy pregnant mothers had a considerably larger fall in DBP and MAP [19]. Our study results were also comparable to their study.

According to Ashok V. et al. a prospective cohort study, the magnitude of the decrease in SBP was comparable in both groups, whereas the magnitude of the decrease in DBP and MAP was significantly smaller in preeclamptic patients [15]. This study contradicted our study findings on SBP changes. This could be due to the baricity of bupivacaine used in hyperbaric therapy, but in our study isobaric therapy was used.

Iranian researchers Nikoosersht et al. (prospective) observed that despite normotensive women receiving more IV fluids, the incidence of hypotension in severely preeclamptic women was found to be much lower (55% versus 89%), (2.5 versus 2.4 L) [37]. Despite differences, this study found a high incidence of hypotension in both groups. The level of sensory block T4 in his study but in our study T5, might explain why the incidence of hypotension was higher in their study.

According to Mendes et al. (Brazil) a prospective cohort study, the mean drop in systolic blood pressure was 27.5 percent in the severely preeclamptic group and 24.2% in the normotensive group, while diastolic blood pressure decreased by 33.1 percent and 35.9 percent, respectively, and this was not statistically significant. They found no statistically significant difference between the two groups in terms of hypotension, ephedrine use, and total ephedrine dose [18]. This study contradicted our study findings. The use of vasodilators in his study and the small sample size might be the causes of the discrepancy.

In Ethiopia, a prospective cohort study by Alemayehu et al. found that the incidence of hypotension was 34.1 percent in preeclamptics compared to 55.6 percent in healthy parturients. This was similar to our research finding, but the magnitude of hypotension in our finding was relatively higher than that of Alemayehu [27]. The discrepancy in the magnitude of hypotension might be due to sampling size and also that the estimated blood loss was high in our study.

Our finding was supported by Tamiru et al. on 84 parturients undergoing cesarean section, which showed a higher incidence of hypotension in the normotensive group compared to the preeclamptic group (31% vs.59.5%, *P* < 0.05) [39]. The defined hypotension with a 30% decline in MAP from baseline vs. 20% in our study could be due to the definition of hypotension as a possible reason for the higher discrepancy magnitude of hypotension.

Following spinal anesthetic induction in both groups, the mean values of HR did not alter significantly during the first 15 min. However, the mean change of heart rate from 18 min to the end of the surgery showed a significant difference between the groups with a *p*- value less than 0.05. This result was contradicting previous work [19, 28, 36]. This might be due to vasopressor consumption in our study.

The difference in hypotension incidence is caused by factors connected to preeclampsia. The possibility is that chronic vasoconstriction is partially caused by damaged vascular endothelium, as observed in preeclampsia, which produces more endogenous vasopressors like thromboxane and endothelia [40].

In contrast to normal pregnancy, where the altered vascular tone, decreased response to endogenous vasopressors, and increased synthesis of vasodilator prostaglandins and nitric oxide make them particularly sensitive to spinal anesthesia and cause hypotension after spinal anesthesia, this phenomenon does not change after SA in preeclampsia, resulting in fewer hemodynamic changes [41]. Strength our research was conducted as a prospective multicenter cohort study. No loss to follow-up of patients, which results in missing data. Limitations of the study, Antihypertensive, intraoperative blood loss, intraoperative fluid consumption, and duration of surgery were not controlled. Lack of invasive blood pressure measurement.

## Conclusions

In contrast to normotensive parturients undergoing caesarean section under spinal anaesthesia, our study found that the hemodynamic change was lower in preeclamptic parturients. The proportion of preeclamptic women who develop hypotension was 47%, compared to 74% of normotensive parturients (Fig. 5).

## Data Availability

All data supporting this manuscript are available in this published article.

## Abbreviations

ASA: American Society of Anesthesiology
ANOVA: Analysis of Variance
BMI: Body Mass Index
CI: Confidence Interval
CS: Caesarean Section
DB: Diastolic Blood Pressor
ECG: Electrocardiograph
HR: Heart Rate
IQR: Interquartile Range
IU: International Unit
IV: Intravenous
KGH: Kuyu General Hospital
MAP: Mean Arterial Pressure
NIBP: Noninvasive Automated Blood Pressure
RR: Relative Risk
SA: Spinal Anesthesia
SB: Systolic Blood presser
SD: Standard Deviation
SPSS: Statistical Package for the Social Sciences
SUCSH: Salale University Comprehensive Specialized Hospital
